# Biosecurity deficiencies in HPAI-affected poultry farms in Korea, 2020/2021–2024/2025 seasons

**DOI:** 10.3389/fvets.2026.1863522

**Published:** 2026-07-17

**Authors:** Hachung Yoon, Kyoungsook Kim, Keesung Hong

**Affiliations:** Veterinary Epidemiology Division, Animal and Plant Quarantine Agency, Gimcheon, Republic of Korea

**Keywords:** association rule analysis, biosecurity deficiencies, cluster analysis, highly pathogenic avian influenza (HPAI), Korea, outbreak investigation, poultry farms

## Abstract

**Introduction:**

Highly pathogenic avian influenza (HPAI) remains a persistent threat to poultry production systems despite reinforced biosecurity measures. This study aimed to characterize biosecurity deficiencies in HPAI-affected farms in Korea and to identify structured patterns of deficiencies.

**Methods:**

Outbreak investigation reports from 311 HPAI-affected farms (2020/2021–2024/2025 seasons) were analyzed, yielding 3,172 biosecurity deficiency records classified into 16 categories across three operational domains. Frequency analysis, *k*-means clustering, and association rule analysis were applied to identify deficiency patterns and co-occurrence structures.

**Results:**

Wildlife control was the most frequent deficiency (85.9%), followed by operational management (65.9%) and deficiencies related to anteroom biosecurity measures in barns (56.3%). Four distinct farm-level deficiency profiles were identified, with significant differences in cluster membership between chicken and duck farms. Association rule analysis revealed consistent co-occurring deficiency pairs, particularly involving farm-entry measures such as visitor and vehicle disinfection.

**Discussion:**

These findings indicate that biosecurity deficiencies are not isolated but form structured and interconnected patterns. A system-level approach incorporating targeted, pattern-based biosecurity interventions and risk-based surveillance prioritization may improve the sustainability and effectiveness of HPAI prevention and control in poultry production systems.

## 1 Introduction

Highly pathogenic avian influenza (HPAI) H5N1 clade 2.3.4.4b has reshaped the global avian influenza landscape through its wide geographic spread, expanded host range, and substantial impacts on poultry production and food systems worldwide (1, 2). In the Republic of Korea, HPAI is the primary Class I notifiable infectious disease affecting poultry under the Act on the Prevention of Contagious Animal Diseases, requiring the highest level of control and emergency response measures (3). HPAI outbreaks have recurred since 2003, involving multiple subtypes including H5N1, H5N8, and H5N6, and have continued to occur annually in both poultry and wild birds (4, 5). These outbreaks show marked winter seasonality associated with migratory wild birds, with high-density waterfowl aggregation increasing transmission risk (6, 7). In response, the government operates a reinforced winter disease-control period with enhanced biosecurity requirements for poultry farms (8, 9).

Biosecurity comprises management and physical measures aimed at preventing the introduction and spread of infectious diseases within animal populations (10). However, implementation remains inconsistent across poultry farms, with substantial compliance gaps reported in relation to recommended biosecurity practices (11). Practical constraints, including limited training, operational inconvenience, and financial burden, may further hinder effective implementation (12).

Beyond its economic impact on the poultry industry, HPAI is increasingly recognized as an emerging zoonotic disease with critical implications for both public health and food security through spillover into diverse mammalian species, including cattle and humans (13). Given the substantial economic burden of transboundary animal diseases and their potential for large-scale spread, effective control requires integrated strategies combining biosecurity, surveillance, and veterinary response capacity (14–17). As HPAI continues to evolve and spread, control efforts must move beyond single interventions toward adaptive, system-level approaches (17, 18). Failure to implement such strategies risks long-term establishment of HPAI in both wild and domestic systems, with serious implications for global food security (18, 19).

This study provides a system-level characterization of biosecurity deficiencies in poultry farms with confirmed HPAI outbreaks by identifying structured patterns, farm-level deficiency profiles, and co-occurring failure mechanisms. We aimed to move beyond isolated assessments of individual biosecurity measures and instead examine how multiple weaknesses interact within farm biosecurity systems.

## 2 Materials and methods

### 2.1 Data

Outbreak investigation reports of poultry farms with confirmed HPAI outbreaks were retrieved from the publicly available website of the Animal and Plant Quarantine Agency (20). HPAI-affected farms were defined as farms with laboratory-confirmed HPAI outbreaks confirmed by reverse transcription polymerase chain reaction (RT-PCR) and classified as highly pathogenic based on the hemagglutinin (HA) cleavage site and/or intravenous pathogenicity index, following World Organisation for Animal Health (WOAH) guidelines (21). Biosecurity deficiencies were extracted from the reports and classified according to the Act on the Prevention of Contagious Animal Diseases (3) and the public notice on biosecurity standards for poultry farms during the winter reinforced disease-control period (9). A total of 3,172 deficiency records were identified from 311 HPAI-affected farms across five winter seasons (2020/2021–2024/2025), comprising 159 chicken farms, 141 duck farms, and 11 farms raising other poultry species, such as quail and geese. These farms represented all officially reported HPAI outbreak farms publicly available on the APQA website during the study period and were treated as independent farm-level units in the analysis. Deficiencies were grouped into 16 categories across three operational domains (Table 1).

**Table 1:** Classification of biosecurity deficiency categories in HPAI-affected farms, 2020/2021–2024/2025.

Operational domain	Deficiency category	Description
Farm entrance	Vehicle control	• Barriers at entrance • Restricted vehicle access (prohibited vehicle types)
	Vehicle disinfection	• Installation of fixed vehicle disinfection systems • Use of high-pressure spraying targeting undercarriage and wheels
	Biosecurity booth	• Personal protective equipment (PPE; footwear, clothing) • Footbath • Hand sanitizer • Availability of disinfection facility
	Visitor control	• Visitor logbook • Pre-entry declaration for vaccination teams and poultry loading/unloading workers
	Visitor disinfection	• Disinfection using disinfectant spraying or ultraviolet light
	Visitor PPE	• Provision and use of farm-dedicated PPE for visitors
	Secondary entrance	• Application of same biosecurity measures as main entrance
On-farm biosecurity	Farm disinfection practices	• Disinfection of access roads • Disinfection of on-farm areas • Disinfection inside and outside barns • Appropriate use of disinfectants
	Facility footbaths	• Installation of footbaths at key locations (e.g., farm office, egg collection room, feed storage area)
	Equipment management	• Outdoor storage of equipment • Cleaning and disinfection before and after use • Cleaning and disinfection when moving between barns • No sharing of equipment with other farms
	Wildlife control	• Fencing • Avoidance of free-range rearing • Bird-proof netting • Rodent control • On-farm housekeeping • Feed bin base area sanitation
	CCTV monitoring	• Installation of cameras at designated locations • Retention of video records for ≥30 days
	Operational management	• Compliance with biosecurity guidelines • Husbandry/production logbook • Disinfection certificates for visiting vehicles issued by disinfection stations • Facility maintenance • Registration of farm workers (including foreign workers) • Biosecurity training • Facility maintenance; Registration of farm workers (including foreign workers)
Barn biosecurity	Anteroom	• Installation of anteroom • Separation of dirty and clean zones • Footbath • Dedicated boots and work clothing • Hand sanitizer availability
	Rear door	• Application of same biosecurity measures as main barn entrance (anteroom, supplies, disinfection)
	Workers	• Use of anteroom • Dedicated boots • Dedicated work clothing

### 2.2 Frequency analysis

Three complementary metrics were used to summarize biosecurity deficiencies. i) farm-level occurrence was defined as the number and proportion of farms with at least one identified deficiency in a given category, regardless of how many times the deficiency was recorded on the same farm. ii) deficiency record counts represented the total number of recorded deficiency events across all farm inspections and visits, allowing multiple records from the same farm. iii) farm-level deficiency burden was defined as the total number of deficiency records identified per farm. Although farms raising other poultry species were included in the overall summaries, species-specific analyses were restricted to chicken and duck farms due to the limited number of farms representing other poultry species. Differences in deficiency burden between chicken and duck farms were assessed using the Mann–Whitney U test, while differences across clusters were evaluated using the Kruskal–Wallis test.

Farm-level occurrence was calculated as the proportion of HPAI-confirmed farms in which at least one deficiency belonging to the category was identified. The numerator was the number of farms with at least one identified deficiency in the corresponding category, and the denominator was the total number of HPAI-confirmed farms included in the analysis, either overall or within each species group (chicken or duck farms). The presence of at least one deficiency was compared between chicken and duck farms using the chi-square test or Fisher’s exact test, as appropriate, and odds ratios (ORs) with 95% confidence intervals (CIs) were calculated. To account for multiple comparisons, *p*-values were adjusted using the Benjamini–Hochberg false discovery rate (FDR) procedure (22).

### 2.3 Clustering

For each farm, deficiency counts across the 16 categories were aggregated and converted into proportional profiles by dividing each category count by the total number of deficiencies identified on that farm, thereby standardizing for differences in overall deficiency burden. K-means clustering was applied to these 16-category proportional profiles using Euclidean distance (23). Candidate solutions (*k* = 2–10) were evaluated based on the elbow criterion and interpretability of cluster patterns, and a four-cluster solution was selected. All analyses were conducted in Python 3.12.7 (Python Software Foundation, Wilmington, DE, United States) using scikit-learn 1.5.1 (Inria, Paris, France).

### 2.4 Association rule analysis

Association rule analysis was performed at the farm level after converting each biosecurity deficiency category into a binary variable indicating presence or absence. Rules were generated using the Apriori algorithm and evaluated using three standard metrics: support (proportion of farms in which both deficiencies co-occur), confidence (conditional probability of one deficiency given another), and lift (strength of association relative to independence) (24). Thresholds of support ≥ 0.15, confidence ≥ 0.60, and lift > 1.05 were selected based on commonly used criteria in exploratory association rule mining studies and were empirically adjusted according to the dataset size and distribution to retain stable and interpretable co-occurrence patterns while minimizing sparse or spurious associations. Reciprocal rules (i.e., A → B and B → A) were merged into undirected co-occurring pairs, and each pair was characterized by joint support, lift, and the higher of the two directional confidence values.

## 3 Results

### 3.1 Frequency of biosecurity deficiency categories

Table 2 summarizes the number and proportion of farms with at least one identified deficiency in each category (farm-level occurrence). In contrast, Supplementary Table S1 presents the total number of recorded deficiency events across all farm inspections, including repeated records from the same farm. Across all farms, wildlife control was the most commonly identified deficiency (85.9%), followed by operational management (65.9%), anteroom (56.3%), workers (52.7%), vehicle disinfection (52.1%), visitor disinfection (48.2%), and equipment management (46.6%). Species-specific differences were observed in several categories based on chi-square or Fisher’s exact tests. Compared with duck farms, chicken farms more frequently showed deficiencies in visitor control (50.9%), biosecurity booth (34.0%), and rear door measures (24.5%). In contrast, deficiencies related to operational management (75.9%), equipment management (63.1%), and workers (63.1%) were more frequent in duck farms. All of these differences remained significant after FDR correction (Table 2).

**Table 2:** Number and proportion of farms with at least one biosecurity deficiency by category in HPAI-affected farms, 2020/2021–2024/2025.

Operational domain	Deficiency category	All/311 *n* (%)	Chicken/159 *n* (%)	Duck/141 *n* (%)	OR (duck vs. chicken)	95% CI	Adjusted *p*-value (FDR)
Farm entrance	Vehicle control	107 (34.4)	61 (38.4)	42 (29.8)	0.7	0.4–1.1	0.192
	Vehicle disinfection	163 (52.4)	84 (52.8)	74 (52.5)	0.9	0.6–1.5	0.816
	Biosecurity booth	90 (28.9)	54 (34.0)	31 (22.0)	0.5	0.3–0.9	0.039
	Visitor control	129 (41.5)	81 (50.9)	45 (31.9)	0.4	0.3–0.7	0.003
	Visitor disinfection	150 (48.2)	74 (46.5)	71 (50.4)	1.1	0.7–1.7	0.816
	Visitor PPE	130 (41.8)	73 (45.9)	55 (39.0)	0.7	0.5–1.1	0.255
	Secondary entrance	51 (16.4)	31 (19.5)	19 (13.5)	0.6	0.3–1.2	0.237
On-farm biosecurity	Farm disinfection practices	75 (24.1)	41 (25.8)	29 (20.6)	0.7	0.4–1.2	0.338
	Facility footbaths	107 (34.4)	58 (36.5)	47 (33.3)	0.8	0.5–1.3	0.611
	Equipment management	145 (46.6)	53 (33.3)	89 (63.1)	3.2	2.0–5.1	<0.001
	Wildlife control	267 (85.9)	133 (83.6)	123 (87.2)	1.1	0.6–2.1	0.816
	CCTV monitoring	129 (41.5)	67 (42.1)	60 (42.6)	1.0	0.6–1.5	0.897
	Operational management	205 (65.9)	90 (56.6)	107 (75.9)	2.2	1.3–3.6	0.008
Barn biosecurity	Anteroom	175 (56.3)	83 (52.2)	88 (62.4)	1.4	0.9–2.2	0.237
	Rear door	58 (18.6)	39 (24.5)	17 (12.1)	0.4	0.2–0.8	0.013
	Workers	164 (52.7)	70 (44.0)	89 (63.1)	2.0	1.3–3.2	0.009

Each farm was counted once per deficiency category, regardless of repeated observations. Values are presented as *n* (%). The numerator represents the number of HPAI-affected farms with at least one identified deficiency within a given category, whereas the denominator represents the total number of HPAI-affected farms included in the analysis, either overall or within each species group for species-specific comparisons. ORs compare duck farms with chicken farms (reference). *p*-values were obtained using chi-square or Fisher’s exact tests and adjusted by FDR. Analyses were restricted to chicken and duck farms.

Consistent with these findings, ORs indicated higher odds in duck farms relative to chicken farms for operational management (OR = 2.2, 95% CI = 1.3–3.6), equipment management (OR = 3.2, 95% CI = 2.0–5.1), and workers (OR = 2.0, 95% CI = 1.3–3.2), and lower odds for visitor control (OR = 0.4, 95% CI = 0.3–0.7), biosecurity booth (OR = 0.5, 95% CI = 0.3–0.9), and rear door measures (OR = 0.4, 95% CI = 0.2–0.8). However, the overall number of deficiencies per farm was comparable between chicken and duck farms (10.4 ± 5.2 vs. 10.0 ± 4.3, respectively; Mann–Whitney *U* test, *p* = 0.886).

### 3.2 Clustering and farm-level deficiency profiles

Farms were clustered based on the proportional composition of deficiencies across the 16 categories. A four-cluster solution identified distinct and interpretable farm-level biosecurity deficiency profiles: on-farm operational-management–dominant (Cluster 0, *n* = 65), on-farm wildlife-control–dominant (Cluster 1, *n* = 57), barn-focused (Cluster 2, *n* = 96), and entrance-focused (Cluster 3, *n* = 95) (Table 3). Cluster membership differed significantly between chicken and duck farms (*p* = 0.009; Cramér’s V = 0.195), with a higher proportion of duck farms in Cluster 0. Differences in deficiency burden across clusters were also significant (Kruskal–Wallis test, *p* = 7.78 × 10^−9^). *Post hoc* comparisons with FDR correction indicated that Cluster 1 had a lower deficiency burden than Clusters 0, 2, and 3.

**Table 3:** Farm-level biosecurity deficiency cluster profiles and their distribution by poultry species in HPAI-affected farms, 2020/2021–2024/2025.

Cluster	Profile	Dominant characteristics	Deficiency burden per farm	All *n* (%)	Chicken *n* (%)	Duck *n* (%)
Cluster 0	On-farm operational-management–dominant	Operational management with wildlife control and other on-farm biosecurity items	10.5 ± 4.9 (7.0, 10.0, 13.0)	65 (20.8)	21 (13.3)	41 (28.5)
Cluster 1	On-farm wildlife-control–dominant	Wildlife control with additional contributions from CCTV monitoring and operational management	6.9 ± 2.9 (5.0, 6.0, 9.0)	57 (18.2)	32 (20.3)	20 (13.9)
Cluster 2	Barn-focused	Barn anteroom and worker deficiencies with substantial wildlife control and other on-farm components	11.0 ± 4.9 (7.0, 10.0, 14.0)	96 (30.7)	50 (31.6)	44 (30.6)
Cluster 3	Entrance-focused	Farm-entrance deficiencies, particularly visitor PPE and related items	10.9 ± 4.8 (8.0, 11.0, 14.0)	95 (30.3)	55 (34.8)	39 (27.1)

Values are presented as *n* (%) unless otherwise indicated. Deficiency burden is expressed as mean ± SD (Q1, median, Q3). Percentages are based on the number of farms within each species and overall.

### 3.3 Association rule analysis

**Figure 1:**
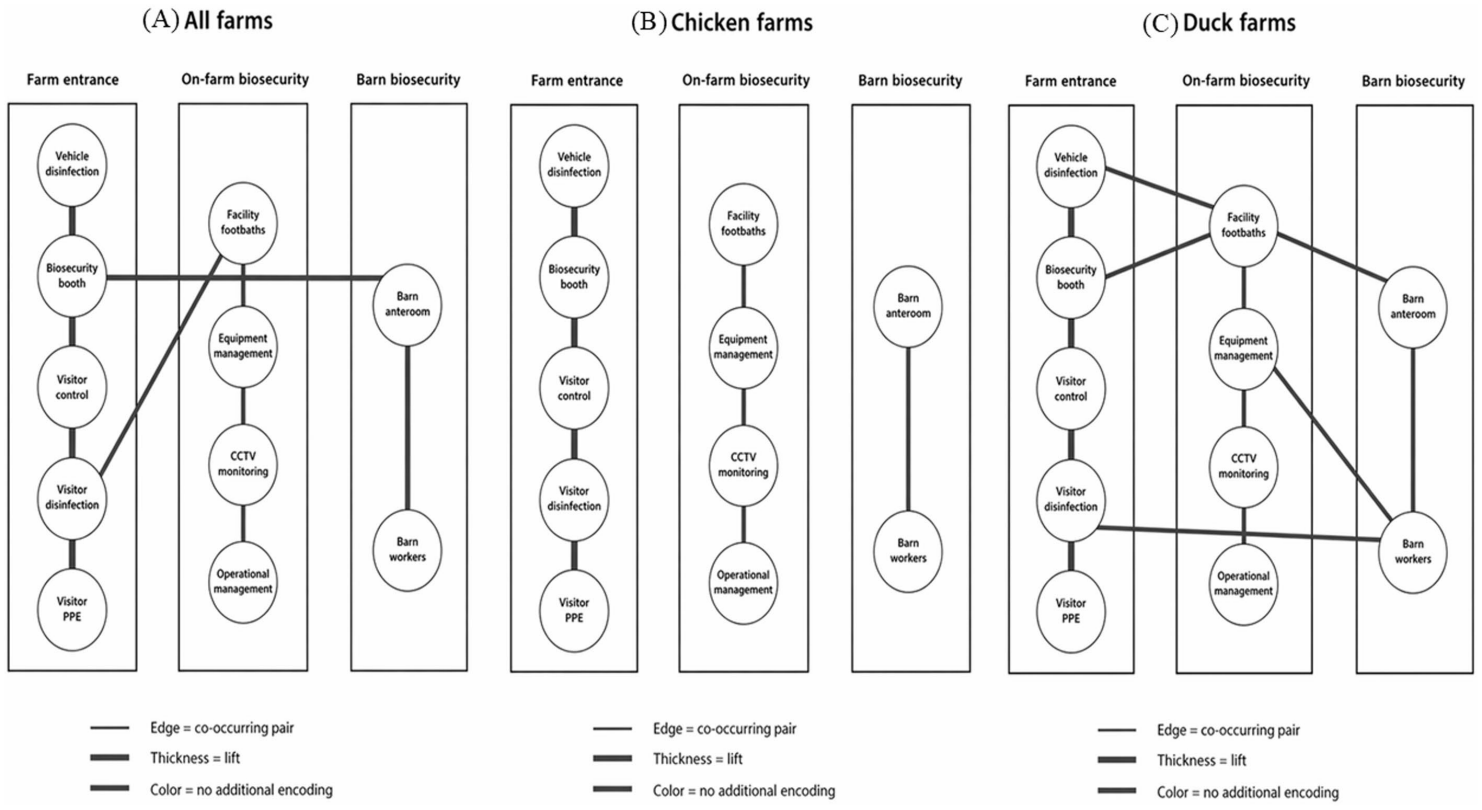
Network visualization of major co-occurring biosecurity deficiency category pairs in HPAI-affected farms, 2020/2021–2024/2025. **(A)** All farms, **(B)** chicken farms, and **(C)** duck farms. Nodes represent deficiency categories, and edges indicate co-occurring pairs. Edge thickness is proportional to lift, and edge labels denote joint support and lift. Only pairs with joint support ≥ 0.20 are displayed.

Association rule analysis identified major co-occurring biosecurity deficiency pairs across all farms and within chicken and duck farms (Table 4). Across all farms, the most prominent co-occurring pair was visitor disinfection and vehicle disinfection, with additional major pairs involving equipment management and operational management, as well as barn workers and barn anteroom. Species-specific patterns were observed. In chicken farms, co-occurring pairs were primarily concentrated around farm-entry and visitor-related measures. In contrast, in duck farms, co-occurring pairs more frequently linked farm-entry and barn-related deficiencies. These patterns are visualized in Figure 1.

**Table 4:** Major co-occurring biosecurity deficiency category pairs in HPAI-affected farms, 2020/2021–2024/2025.

Co-occurring deficiency pair	Joint support (%)	Confidence (%)	Lift
(A) All farms			
Visitor disinfection—vehicle disinfection	37.4	78.0	1.5
Equipment management—Operational management	36.4	78.6	1.2
Barn workers—barn anteroom	34.5	65.9	1.2
Visitor PPE—vehicle disinfection	29.4	70.8	1.4
Visitor PPE—visitor disinfection	27.8	66.9	1.4
Biosecurity booth—barn anteroom	21.4	74.4	1.3
Facility footbaths—visitor disinfection	20.8	60.7	1.3

Co-occurring deficiency pair	Joint support (%)	Confidence (%)	Lift
(B) Chicken farm			
Visitor disinfection—vehicle disinfection	36.1	77.0	1.5
Visitor control—vehicle disinfection	34.2	65.9	1.2
Visitor PPE—vehicle disinfection	32.9	71.2	1.3
Visitor disinfection—visitor control	32.3	68.9	1.3
Barn workers—barn anteroom	30.4	68.6	1.3
Visitor PPE—visitor disinfection	29.1	63.0	1.4
Equipment management—operational management	24.7	73.6	1.3

Co-occurring deficiency pair	Joint support (%)	Confidence (%)	Lift
(C) Duck farms			
Visitor disinfection—vehicle disinfection	38.2	77.5	1.5
Vehicle disinfection—barn workers	38.2	74.3	1.2
Visitor disinfection—barn workers	36.8	74.6	1.2
Vehicle disinfection—barn anteroom	36.8	71.6	1.2
Visitor PPE—visitor disinfection	27.1	70.9	1.4
Visitor PPE—vehicle disinfection	26.4	69.1	1.3
Facility footbaths—barn anteroom	24.3	74.5	1.2
Barn rear door—visitor control	7.6	64.7	2.1

Only major co-occurring pairs are shown. Joint support indicates the proportion of farms with both deficiencies. Confidence is reported for the higher-confidence direction, and lift > 1 indicates positive co-occurrence.

## 4 Discussion

Biosecurity deficiencies in HPAI-affected farms in Korea showed recurring and structured patterns, indicating that failures were not isolated compliance gaps but interconnected systems of deficiencies. This system-level perspective provides a basis for targeted, pattern-based biosecurity interventions and risk-informed control strategies in poultry production systems. The predominance of wildlife-control deficiencies, together with recurrent weaknesses in operational management, barn anterooms, workers, and farm-entry measures, suggests that biosecurity failures in outbreak farms reflect both external exposure at the wild bird–farm interface and internal gaps in routine implementation. This interpretation is consistent with studies showing that structural, operational, and behavioral biosecurity gaps often coexist rather than occur in isolation (25–27). The prominence of wildlife-control deficiencies is epidemiologically plausible given the role of wild birds in HPAI introduction and maintenance. Previous studies have identified proximity to wild bird populations and water bodies as key risk factors (28), and long-term patterns in South Korea link outbreaks to migratory bird introduction followed by farm-level spread (6, 7). Similar weaknesses in wild bird exclusion have also been reported in Europe (25, 29), highlighting the farm perimeter as a critical vulnerability.

Species-specific differences indicate that biosecurity weaknesses may manifest differently across poultry sectors. Duck farms more often showed deficiencies in equipment management, operational management, and workers, whereas chicken farms more often showed deficiencies related to visitor control, biosecurity booths, and rear-door measures. These differences may reflect sector-specific practices, housing structures, and exposure contexts, consistent with previous findings that farm characteristics influence HPAI transmission dynamics (30, 31). In the Republic of Korea, commercial chicken farms are generally operated under highly industrialized enclosed housing systems equipped with mechanical ventilation and evaporative cooling systems, whereas duck farms frequently utilize natural ventilation systems with winch curtains. These structural and management differences may partially explain the greater prominence of operational-management and worker-related deficiencies observed in duck farms, consistent with previous studies highlighting the epidemiological importance of duck production systems in HPAI transmission in Korea (32).

Cluster analysis showed that deficiencies accumulated in distinct farm-level profiles rather than as isolated checklist failures. The identified patterns support viewing farm biosecurity as a system of interacting practices shaped by structural and behavioral constraints (33, 34). Classifying farms according to dominant deficiency profiles may therefore provide more informative guidance for intervention design than relying on aggregate scores alone (35). These profile-level patterns further suggest that individual deficiencies may not occur independently, but rather emerge as interconnected combinations of weaknesses within farm biosecurity systems. Association-rule analysis showed that biosecurity deficiencies were interconnected, particularly between visitor and vehicle disinfection and other linked entry and management measures. Studies in Korea and Europe similarly reported that hygiene practices were associated with reduced HPAI risk, while routine operational breaches remained common (36, 37). These findings suggest that biosecurity effectiveness depends on coordinated system-level implementation rather than isolated measures (24). Structured biosecurity may therefore improve sustainability, consistency, and cost-effectiveness by reducing cumulative vulnerabilities across transmission pathways and by complementing surveillance, rapid detection, movement control, and emergency response strategies.

Together, these findings indicate that biosecurity deficiencies in HPAI-affected farms are structured, sector-specific, and tend to cluster into interpretable farm-level patterns. Recurrent co-occurring deficiency pairs suggest that weaknesses accumulate in consistent combinations, particularly around farm-entry measures. This supports a shift from single-item compliance checks toward integrated, system-level approaches to biosecurity. From a practical perspective, the identified deficiency profiles may support risk-based prioritization of biosecurity interventions according to farm characteristics and dominant vulnerability patterns. Farms with entrance-focused profiles may benefit from strengthened visitor control and disinfection measures, whereas barn- or management-dominant farms may require improvements in worker practices, equipment handling, and operational oversight. Training and technology-assisted monitoring, including CCTV, may further enhance compliance, particularly for behaviorally demanding procedures (27, 36, 38). Collectively, these findings may help inform more targeted surveillance strategies and adaptive biosecurity policies in poultry production systems.

This study is limited to farms with confirmed HPAI outbreaks, and the identified patterns do not establish direct causality. However, these deficiencies represent conditions that may contribute to disease introduction and spread, and their consistent implementation remains essential for effective HPAI prevention.

## Data Availability

The raw data supporting the conclusions of this article will be made available by the authors.
