# Supplementary material for: Biosecurity deficiencies in HPAI-affected poultry farms in Korea, 2020/2021–2024/2025 seasons
Source: Front Vet Sci. 2026 Jul 17;13:1863522. doi: 10.3389/fvets.2026.1863522 (PMC13423691; doi:10.3389/fvets.2026.1863522)
Supplement: Supplementary file 1 [file Table_1.DOCX]

Supplementary Material

# Supplementary Tables

Table S1.

Number of biosecurity deficiency records by category in HPAI-affected farms, 2020/2021–2024/2025

| **Operational domain** | **Deficiency category** | **All**  **n** | **Chicken**  **n** | **Duck**  **n** |
| --- | --- | --- | --- | --- |
| Farm entrance | Vehicle control | 138 | 79 | 53 |
|  | Vehicle disinfection | 172 | 89 | 77 |
|  | Biosecurity booth | 137 | 89 | 43 |
|  | Visitor control | 147 | 99 | 45 |
|  | Visitor disinfection | 157 | 78 | 74 |
|  | Visitor PPE | 238 | 139 | 96 |
|  | Secondary entrance | 77 | 49 | 27 |
| On-farm biosecurity | Farm disinfection practices | 81 | 44 | 32 |
|  | Facility footbaths | 130 | 77 | 51 |
|  | Equipment management | 185 | 57 | 124 |
|  | Wildlife control | 575 | 303 | 244 |
|  | CCTV monitoring | 144 | 75 | 66 |
|  | Operational management | 370 | 162 | 194 |
| Barn biosecurity | Anteroom | 294 | 144 | 142 |
|  | Rear door | 60 | 41 | 17 |
|  | Workers | 267 | 110 | 148 |

**Note:** All records include 11 farms raising other poultry species (e.g., quail and geese).

**
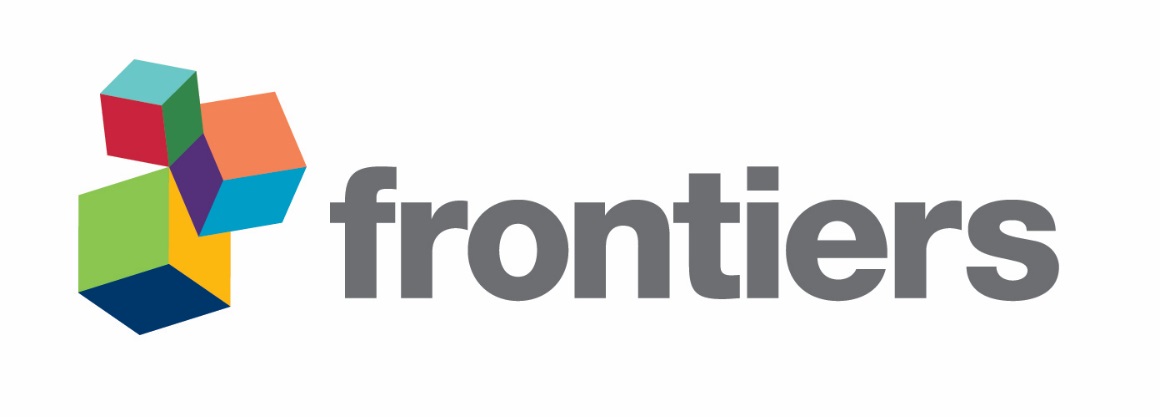
**
